# Reforming cap training in latvia: Nowhere to go but up

**DOI:** 10.1192/j.eurpsy.2021.193

**Published:** 2021-08-13

**Authors:** N. Bezborodovs

**Affiliations:** Department Of Psychiatry And Narcology, Riga Stradins University, Riga, Latvia

**Keywords:** CAP training, child and adolescent psychiatry, Residency, Recruitment

## Abstract

There is still substantial variation in the amount, structure and quality of child and adolescent psychiatry (CAP) training across European countries, both in the training process of general adult psychiatry and CAP specialists. Inconsistency, scarcity and low quality of CAP exposure has been consistently identified by psychiatric trainees as one of major issues in organization of training. In the decades of independence, following the collapse of the Soviet Union, Latvia has witnessed a gradual decline in the number of CAP specialists in the country due to chronically low recruitment rates, that has subsequently led to a critical human resource deficit in the field, and rapid deterioration of availability and quality of CAP care. Only since the year 2018, when the normative regulation, structure and contents of CAP training in Latvia have been significantly reformed, there was a change in recruitment trends, that gives hope for resolution of the human resource crisis in the CAP field in the years to come. In this talk the author will share his experience of redesigning the CAP training program in Latvia, and discuss the motivations, challenges and successes one might face while trying to improve CAP training in a particular European country.

**Disclosure:**

No significant relationships.

